# Exendin-4 Inhibits Hepatic Lipogenesis by Increasing β-Catenin Signaling

**DOI:** 10.1371/journal.pone.0166913

**Published:** 2016-12-01

**Authors:** Mi Hae Seo, Jinmi Lee, Seok-Woo Hong, Eun-Jung Rhee, Se Eun Park, Cheol Young Park, Ki Won Oh, Sung Woo Park, Won-Young Lee

**Affiliations:** 1 Department of Internal Medicine, Soonchunhyang University Gumi Hospital, Gumi, Korea; 2 Institute of Medical Research, Kangbuk Samsung Hospital, Sungkyunkwan University School of Medicine, Seoul, Korea; 3 Department of Internal Medicine, Kangbuk Samsung Hospital, Sungkyunkwan University School of Medicine, Seoul, Korea; Universite du Quebec a Montreal, CANADA

## Abstract

The aim of this study is to investigate whether the beneficial effect of exendin-4 on hepatic steatosis is mediated by β-catenin signaling. After the HepG2 human hepatoma cells were treated with PA for 24 hours, total triglycerides levels were increased in a dose-dependent manner, and the expression levels of perilipin family members were upregulated in cells treated with 400 μM PA. For our in vitro model of hepatic steatosis, HepG2 cells were treated with 400 μM palmitic acid (PA) in the presence or absence of 100 nM exendin-4 for 24 hours. PA increased the expression of lipogenic genes, such as sterol regulatory element-binding protein 1c (SREBP-1c), peroxisome proliferator-activated receptor gamma (PPARγ), stearoyl-CoA desaturase 1 (SCD1), fatty acid synthase (FAS), and acetyl-CoA carboxylase (ACC) and triglyceride synthesis-involved genes, such as diacylglycerol acyltransferase 1 (DGAT1) and diacylglycerol acyltransferase 2 (DGAT2) in HepG2 cells, whereas exendin-4 treatment significantly prevented the upregulation of SREBP-1c, PPARγ, SCD1, FAS, ACC, DGAT1 and DGAT2. Moreover, exendin-4 treatment increased the expression of phosphorylated glycogen synthase kinase-3 beta (GSK-3β) in the cytosolic fraction and the expression of β-catenin and transcription factor 4 (TCF4) in the nuclear fraction. In addition, siRNA-mediated inhibition of β-catenin upregulated the expression of lipogenic transcription factors. The protective effects of exendin-4 on intracellular triglyceride content and total triglyceride levels were not observed in cells treated with the β-catenin inhibitor IWR-1. These data suggest that exendin-4 treatment improves hepatic steatosis by inhibiting lipogenesis via activation of Wnt/β-catenin signaling.

## Introduction

Nonalcoholic fatty liver disease (NAFLD) is defined as a spectrum of conditions characterized histologically by hepatic steatosis [1]. The main characteristic of hepatic steatosis is the accumulation of excessive triacylglycerols (TAGs) in hepatocytes [1]. While the precise cellular mechanisms driving the development of hepatic steatosis in humans have not yet been fully elucidated, enhanced activity of the lipogenic pathway is likely to contribute to the development of this disease [2]. In particular, the expression of various lipogenic genes (ACC/acetyl-CoA carboxylase, FAS/fatty acid synthase, SCD1/stearoyl-CoA desaturase 1) is coordinated by key transcriptional regulators (SREBP-1c, sterol regulatory element binding protein-1c; ChREBP, carbohydrate responsive element-binding protein) [3]. Hepatic FFAs derived from three sources, including de novo lipogenesis, plasma free fatty acids or dietary intake, can be re-esterified with glycerol to produce triglyceride and it is stored in lipid droplets.

Exenatide (exendin-4, Ex-4), a glucagon-like peptide-1 (GLP-1) receptor agonist, shares 53% sequence similarity with native GLP-1 [4]. GLP-1R agonists are known to induce multiple signaling pathways intrinsic to β-cell function and hepatic lipogenesis [5,6]. Moreover, exendin-4 has been reported to significantly reduce hepatic steatosis in *ob/ob* mice [7,8]. Gupta *et al*. found that exendin-4 activated important signaling molecules downstream of insulin receptor substrate 1 (IRS-1) in hepatocytes [7]. Furthermore, inhibition of endoplasmic reticulum (ER) stress in obese rodents resulted in decreased SREBP-1c activation and lipogenesis, thereby improving hepatic steatosis [9]. Recently, exendin-4 was reported to improve fatty liver disease by increasing the level of sirtuin (SIRT)-1 mediated fibroblast growth factor (FGF)-21 both *in vivo* and *in vitro* [10].

Numerous studies have reported that Wnt signaling is linked to a variety of human diseases, including obesity, type 2 diabetes (T2 DM), and other metabolic diseases [11–14]. Kawai *et al*. showed that Wnt/β-catenin signaling suppresses adipogenesis by inhibiting C/EBPα-induced PPARγ expression [15]. The variant of transcription factor7-like 2 (TCF7L2; also called T-cell factor 4 or TCF 4), a component of the Wnt signaling pathway, is associated with T2 DM in diverse populations [16,17]. Moreover, Liu *et al*. found that GLP-1 agonists activated TCF7L2-dependent Wnt signaling in isolated mouse pancreatic islets and induced β-cell proliferation [18]. Another study in skeletal muscle cells reported that the activation of Wnt/β-catenin signaling increased insulin sensitivity through reciprocal regulation of Wnt10b and SREBP-1c [19].

However, to the best of our knowledge, a role for Wnt/β-catenin signaling in exendin-4-mediated protection against hepatic lipogenesis has not been described. Here we tested the hypothesis that exendin-4-mediated activation of β-catenin signaling plays a crucial role in the inhibition of hepatic lipogenesis.

## Materials and Methods

### Cell culture and pharmacological treatments

The HepG2 and Huh7 human hepatoma cell lines was purchased from American Type Culture Collection (ATCC, Manassas, VA, USA) and Korean Cell Line Bank (KCLB, Seoul, Korea), respectively. Cells were cultured in Dulbecco’s modified Eagle’s medium (DMEM) (high glucose; Gibco, Grand Island, NY, USA) supplemented with 10% fetal bovine serum and 1% penicillin/streptomycin. The normal mouse hepatocyte cell line (AML12) was purchased from ATCC and was maintained in DMEM/F12 medium (Gibco, Grand Island, NY, USA) supplemented with insulin-transferrin-selenium supplement (100×, liquid) (Gibco, Grand Island, NY, USA), 40ng/ml dexamethasone, 10% fetal bovine serum and 1% penicillin/streptomycin. Cells were seeded in 6-well plates at 2.5 × 10^5^ cells/well and maintained at 37℃ with 5% CO_2_. For the in vitro model of hepatic steatosis, HepG2 cells were treated with 200–500 μM PA (Sigma-Aldrich). We chose the time-point of 24 hours based on TG content (data not shown). For pharmacological treatments, cells grown in the presence of 400 μM PA for 24 hours were treated with or without 100 nM exendin-4 (Sigma-Aldrich) and 10 μM IWR-1 (β-catenin inhibitor; Sigma-Aldrich).

### siRNA transfection

For siRNA-mediated gene silencing, Stealth RNAi siRNA duplexes targeting β-catenin (10620319 and 10620318) or Stealth RNAi negative control duplexes (Invitrogen) were transfected into human HepG2 cells using Lipofectamine RNAiMAX transfection reagent (Invitrogen) according to the manufacturer’s instructions. Cells were cultured under normal growth conditions (37℃, 5% CO_2_) for 24 hours.

### Quantitative real-time RT-PCR

Total RNA was isolated from cells using Trizol reagent (Invitrogen). Reverse transcription reactions were performed using 2 μg total RNA, moloney murine leukemia virus reverse transcriptase (MMLV-RT), and oligo (dT) 12–18 primers (Invitrogen) according to the manufacturer’s instructions. Gene expression levels were analyzed by real-time PCR on a Light-Cycler 480 system (Roche, Lewis, UK) using SYBR Green (Roche) and gene-specific primers (Table 1). (Bioneer Co., Daejeon, Korea). Thermocycling parameters included denaturation at 94°C for 15 seconds, annealing at 55°C for 10 seconds, and extension at 72°C for 20 seconds. Results were normalized to β-actin as an internal control and relative expression levels were calculated using the comparative Ct method (2^delta delta Ct).

**Table 1:** List of human gene primers used for quantitative PCR.

Gene	Forward sequence (5’→3’)	Reverse sequence (5’→3’)	Size (bp)
SREBP-1c	GGCTCCTGCCTACAGCTTCT	CAGCCAGTGGATCACCACA	109
PPARγ	GACCTCAGACAGATTGTCAC	AGTCCTTGTAGATCTCCTGC	106
SCD1	CACCACATTCTTCATTGATTGCA	ATGGCGGCCTTGGAGACT	75
FAS	TATGCTTCTTCGTGCAGCAGTT	GCTGCCACACGCTCCTCTAG	94
ACC	CAGAAGTGACAGACTACAGG	ATCCATGGCTTCCAGGAGTA	125
DGAT1	AACTGGTGTGTGGTGATGCT	CCTTCAGGAACAGAGAAACC	112
DGAT2	CTACAGGTCATCTCAGTGCT	GAAGTAGAGCACAGCGATGA	120
β-catenin	GCAAGCTCATCATACTGGCT	CTTGCATTCCACCAGCTTCT	165
PLIN1	GATCATGAGGACCAGACAGA	CTGCTACCTCACTGAACTTG	92
PLIN2	ACAGACCATTTCTCAGCTCCAT	TATCCAATGCTCCTTTTCCACT	141
PLIN3	GAACAGAGCTACTTCGTACG	CAGTTTCCATCAGGCTTAGG	151
β-actin	TCATGAAGATCCTCACCGAG	CATCTCTTGCTCGAAGTCCA	116

### Western blotting

Nuclear and cytoplasmic extracts were prepared from liver tissue and cultured cells using a commercially available nuclear extraction kit (Cayman Chemical Co., Ann Arbor, MI, USA). Twenty micrograms of protein was resolved on a 4%-12% Bis-Tris NuPAGE gel and transferred to a polyvinylidene difluoride (PVDF) membrane using an iBlot Transfer Stack (Invitrogen). After protein transfer, the following primary antibodies were used: anti-phospho-GSK-3β (#9323; Cell Signaling Technology, Danvers, MA, USA), anti-GSK-3β (#9315; Cell Signaling Technology, Danvers, MA, USA), anti-β-catenin (#9582; Cell Signaling Technology, Danvers, MA, USA), anti-TCF4 (#2569; Cell Signaling Technology), anti-PPARγ (#2443; Cell Signaling Technology), anti-SREBP-1 (sc-366; Santa Cruz Biotechnology, Inc., Santa Cruz, CA, USA), anti-β-actin (#4967; Cell Signaling Technology), and anti-Lamin B1 (ab16048; Abcam, Cambridge, MA, USA). Membranes were then incubated with horseradish peroxidase-conjugated secondary antibodies. Immunoreactive bands were detected by chemiluminescence (GE Healthcare, Piscataway, NJ, USA).

### Lipid detection

Total TGs were extracted from harvested HepG2 cells. Lipid analysis was carried out with a Serum Triglyceride Determination Kit (TR0100, Sigma-Aldrich Corp.) following the manufacturer’s instructions. Protein concentrations were determined with a BCA protein assay kit (Thermo Scientific, Rockford, IL, USA). TG values were normalized to cellular protein contents. Intracellular lipids were visualized by staining with Oil Red O. Briefly, cultured cells were washed 3 times with PBS, fixed in formalin (10%) for 30 min, stained with Oil Red O solution for 1 hour, and washed with distilled water. Images were captured at 400x magnification. To quantitate the intracellular lipid contents, Oil Red O was extracted from each well with 1 ml of isopropanol. Plates were incubated for 5 min, and the resultant absorbances were read spectophotometrically at 540 nm.

### Statistical analysis

All statistical analyses were performed using PASW Statistics 17 (SPSS, Chicago, IL, USA). Data are presented as means ± standard errors. To evaluate the significance of differences between the mean values of different experimental groups, the one-way analysis of variance (ANOVA) test was used. Multiple comparisons between experimental groups were adjusted with the Bonferroni correction. A *p* value <0.05 was considered statistically significant unless otherwise indicated.

## Results

### Palmitic acid induces lipid accumulation in hepatocytes

**Fig 1:**
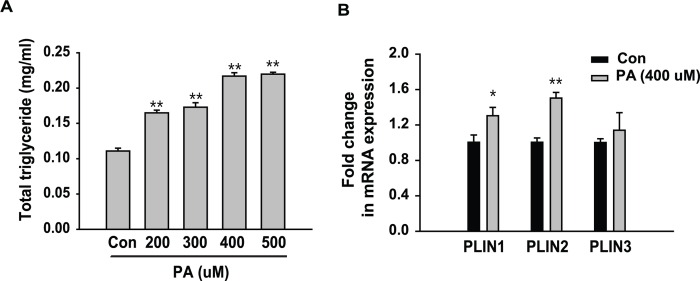
Palmitic acid stimulates lipid accumulation in hepatocytes. (A) HepG2 cells were treated with 200–500 μM palmitic acid (PA) for 24 hours. Triglycerides were extracted from cultured cells and quantitated by enzymatic assays. Triglyceride levels were normalized to total cellular protein contents. (B) HepG2 cells were treated with 400 μM PA for 24 hours. The mRNA expression levels of perilipin 1 (PLIN1), perilipin 2 (PLIN2) and perilipin 3 (PLIN3) were normalized to the level of β-actin. All values are expressed as the mean ± SE (n = 6). * p<0.05, ** p<0.01 compared with control cells.

HepG2 human hepatoma cells were treated with different concentrations of palmitic acid (PA). As shown in Fig 1A, PA treatment increased the levels of intracellular TGs in a dose-dependent manner. We also observed the gene expression of perilipin 1 (PLIN1), perilipin 2 (PLIN2) and perilipin 3 (PLIN3), which are lipid droplet-binding proteins. In cells treated with 400 μM PA, the expression of PLIN1 and PLIN2, but not PLIN3, were significantly increased (Fig 1B). This finding suggests that PA induces steatosis in hepatocytes.

### Exendin-4 inhibits PA-mediated lipogenesis and TG synthesis in hepatocytes

**Fig 2:**
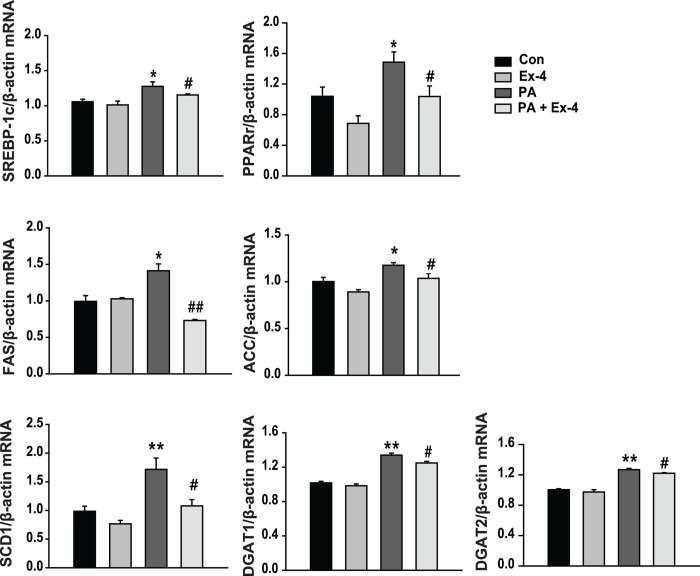
Exendin-4 inhibits palmitic acid-induced hepatic lipogenesis and TG synthesis. HepG2 cells were treated with palmitic acid (PA; 400 μM) either with or without exendin-4 (Ex-4; 100 nM) for 24 hours. The mRNA expression levels of SREBP-1c, PPARγ, SCD1, FAS, ACC, DGAT1, and DGAT2 were normalized to the level of β-actin. All values are expressed as the mean ± SE (n = 6). * p<0.05, ** p<0.01 compared with control cells; # p<0.05, ## p<0.01 compared with PA-treated cells.

To investigate whether exendin-4 affects the expression of genes related to hepatic lipogenesis, HepG2 cells were pretreated with PA and then incubated with exendin-4. The mRNA expression levels of lipogenesis-associated genes, such as SREBP-1c, PPARγ, SCD1, FAS, and ACC, and fatty acid esterification-associated genes, such as diacylglycerol acyltransferase 1 (DGAT1) and diacylglycerol acyltransferase 2 (DGAT2) were increased in PA-treated cells compared with untreated controls. However, exendin-4 treatment prevented the PA-induced upregulation of SREBP-1c, PPARγ, SCD1, FAS, ACC, DGAT1, and DGAT2 expression (Fig 2). Therefore, these data suggest that exendin-4 inhibits PA-mediated hepatic lipogenesis and TG synthesis.

### Exendin-4 promotes β-catenin signaling

**Fig 3:**
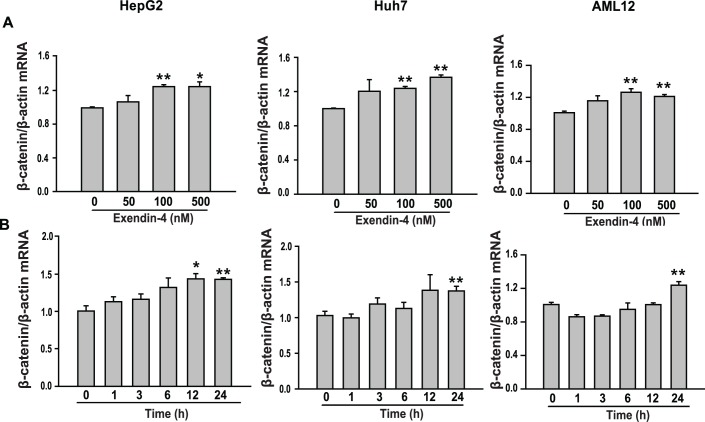
Dose-dependent and time-dependent effects of exendin-4 on β-catenin expression in hepatocytes. (A) HepG2, Huh7 and AML12 cells were treated with three different concentrations (50–500 nM) of exendin-4 for 24 hours. (B) HepG2, Huh7 and AML12 cells were treated with 100 nM exendin-4 for different lengths of time up to 24 hours. β-catenin expression was measured using quantitative (real-time) PCR and normalized to β-actin as a control. All values are expressed as the mean ± SE (n = 6). * p<0.05, ** p<0.01 compared with control cells.

**Fig 4:**
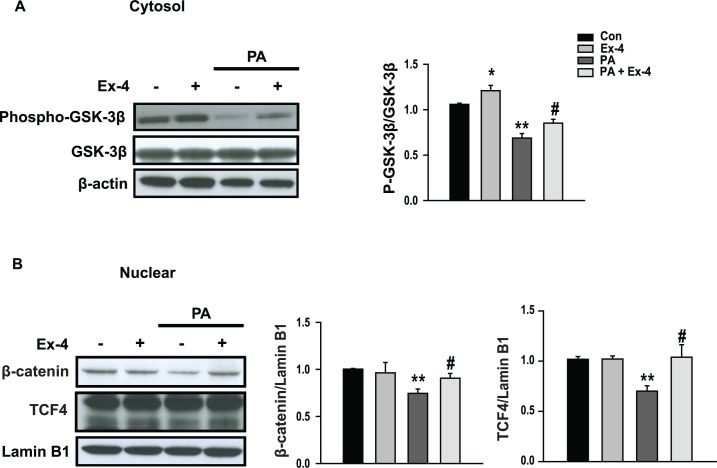
Exendin-4 induces active β-catenin signaling in an in vitro model of steatosis. Cytosolic and nuclear extracts were prepared from HepG2 cells treated with palmitic acid (PA; 400 μM) either with or without exendin-4 (Ex-4; 100 nM) for 24 hours. The levels of (A) cytosolic phosphorylated GSK-3β, (B) nuclear β-catenin and TCF4 were analyzed by western blotting. All values are expressed as the mean ± SE (n = 6). * p<0.05, ** p<0.01 compared with control cells; # p<0.05, ## p<0.01 compared with PA-treated cells.

To investigate whether the actions of exendin-4 were mediated by β-catenin, HepG2, Huh7 and AML12 cells were treated with different concentrations of exendin-4 for 24 hours or were treated with exendin-4 (100 nM) for various time periods. The expression of β-catenin mRNA was increased in a dose-dependent and time-dependent manner by exendin-4 treatment (Fig 3A and 3B). Inactivation of glycogen synthase kinase-3β (GSK-3β) by phosphorylation enhances nuclear accumulation of beta-catenin [14]. In HepG2 cells, the inactive form of GSK-3β, which is phosphorylated at serine-9, was increased in cells treated with PA+exendin-4 compared with PA-treated cells (Fig 4A). Moreover, the expression levels of nuclear β-catenin and transcription factor 4 (TCF4), a central transcription factor in β-catenin signaling, were decreased in cells treated with PA compared with the respective controls. However, exendin-4 treatment reduced these effects (Fig 4B). Consistently, these effects of exendin-4 were observed also in mouse hepatocytes (S1 Fig).

### β-catenin inhibits hepatic lipogenesis

**Fig 5:**
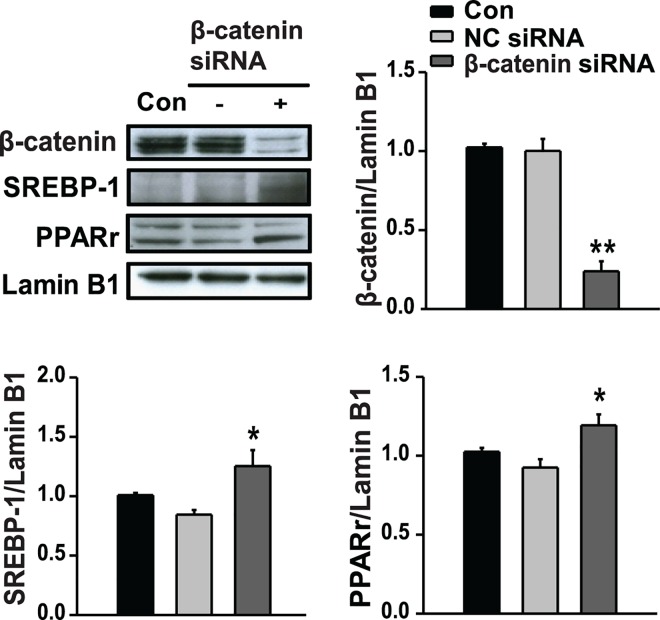
β-catenin downregulates the expression of lipogenic transcription factors. HepG2 cells were transfected with 10 nM siRNA directed against β-catenin for 24 hours. The protein levels of nuclear β-catenin, SREBP-1, and PPARγ were detected by western blot analysis. All values are expressed as the mean ± SE (n = 6). * p<0.05, ** p<0.01 compared with negative control (NC) siRNA-transfected cells.

Regulatory effect of β-catenin on the expression of SREBP-1 and PPARγ, two key regulators of *de novo* lipogenesis, was next assessed by western blotting. The expression levels of nuclear SREBP-1 and PPARγ proteins were significantly increased in cells transfected with β-catenin siRNA compared with negative control siRNA-transfected cells (Fig 5). These results suggest that β-catenin could regulate hepatic lipogenesis.

### Exendin-4 reduces PA-induced lipid accumulation in HepG2 cells via β-catenin signaling

**Fig 6:**
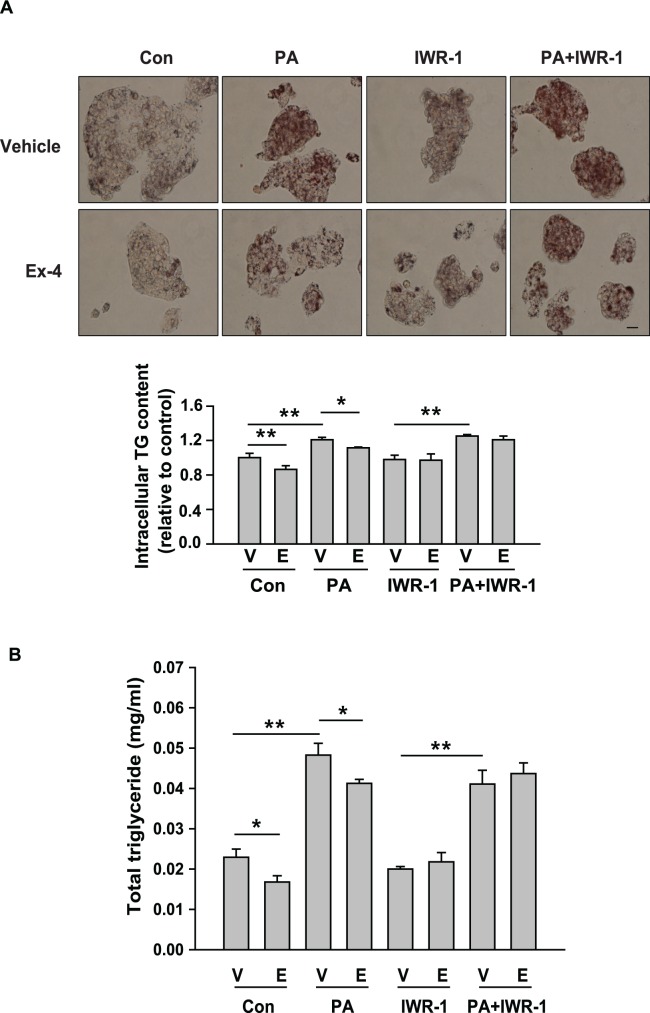
Exendin-4 reduces PA-induced lipid accumulation via β-catenin signaling in HepG2 cells. Cells treated with 400 μM PA were stimulated with 100 nM exendin-4 in the absence or presence of 10 μM IWR-1 for 24 hours. (A) Cells were stained with Oil Red O and images were captured under a light microscope (magnification, 400x). Scale bars = 100 μm. The absorbance of the extracted dye was measured at 540 nm. (B) Triglycerides were extracted from cultured cells and quantitated by enzymatic assays. Triglyceride levels were normalized to total cellular protein contents. All values are expressed as the mean ± SE (n = 6). * p<0.05, ** p<0.01 compared with control cells.

Lipid accumulation in HepG2 cells was next examined by Oil Red O staining. Compared with untreated cells, PA-treated cells exhibited increased lipid accumulation; however, exendin-4 treatment reduced this increase (Fig 6A). However, in cells pretreated with the β-catenin inhibitor IWR-1, this effect of exendin-4 was not observed. Also, total triglyceride levels were consistent with lipid content quantified by oil red o staining (Fig 6B). These results suggest that the protective effect of exendin-4 against PA-induced lipid accumulation is mediated by β-catenin.

## Discussion

**Fig 7:**
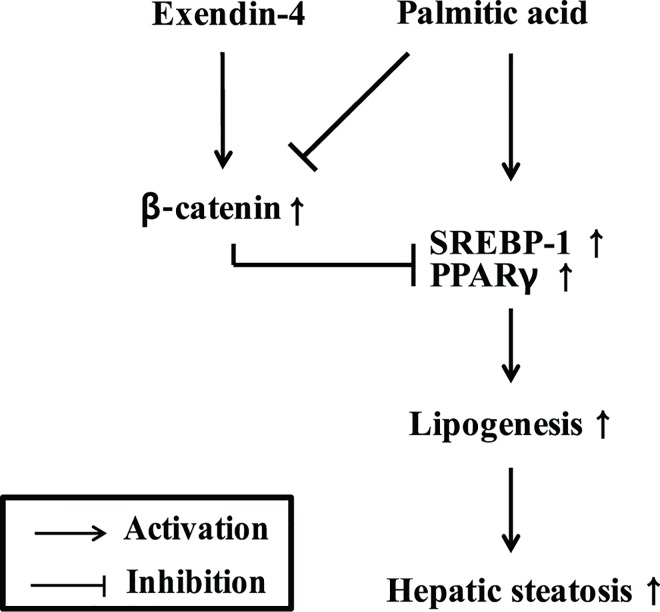
Schematic diagram illustrating a possible mechanism by which exendin-4-driven β-catenin signaling alleviates PA-induced hepatic steatosis.

Exendin-4 has been reported to reduce lipid accumulation in the liver of a mouse model of HF-induced obesity and to improve hepatic lipid metabolism via the sirt1 signaling cascade [20]. In this study, we demonstrated that exendin-4 attenuates hepatic lipogenesis by activating β-catenin. Exendin-4 decreased the expression levels of hepatic SREBP-1 and PPARγ in PA-treated HepG2 cells. Increased expression levels of phospho-GSK-3β, β-catenin and TCF4 induced by exendin-4 treatment were observed in an *in vitro* model of steatosis. However, siRNA-mediated knockdown of β-catenin increased the expression of lipogenic transcription factors and the effect of exendin-4 on PA-induced lipid accumulation was impaired upon treatment with the β-catenin inhibitor IWR-1. These findings suggest that the protective effect of exendin-4 against hepatic lipogenesis is mediated by the Wnt/β-catenin signaling pathway (Fig 7).

Hepatic steatosis is associated with the alteration of nuclear receptors, membrane transport proteins, and cellular enzymes [21]. Hepatic steatosis also includes the *de novo* synthesis of fatty acids from acetyl-CoA or malonyl-CoA, which are further metabolized to TAGs [22]. Fatty acid synthesis is catalyzed by acetyl-coA carboxylase (ACC) and fatty acid synthase (FAS). These enzymes are upregulated by SREBP-1c, lipogenic transcription factor [9,22]. In addition, PPARγ, a lipid-activated transcription factor, is also involved in lipid metabolism and glucose homeostasis [23]. In clinical and animal studies, rosiglitazone and pioglitazone, two PPARγ-selective agonists, have been reported to improve NAFLD and insulin resistance [24,25]. Inoue *et al*. reported that PPARγ expression is increased in the liver by high fat diet in mice [26]. In our previous study, we found that PA induces the nuclear translocation of SREBP-1 [27]. Also, the present study demonstrated that PA increases the expression levels of lipogenic genes, such as SREBP-1, PPARγ, SCD1, FAS, and ACC and fatty acid esterification genes, such as DGAT1 and DGAT2 in human hepatocytes. This finding suggests that PA induces hepatic steatosis by upregulating the expression of lipogenesis and fatty acid esterification-involved genes.

β-catenin is a key protein in the canonical Wnt signaling cascade [28]. In the absence of Wnts, cytoplasmic β-catenin is recruited to a degradation complex containing GSK-3β and adenomatous polyposis coli (APC). This recruitment is followed by β-catenin phosphorylation, ubiquitination, and degradation. Alternatively, Wnt binding to frizzled (FZD) receptors and LRP5/6 coreceptors results in destabilization of this degradation complex, leading to phosphorylation/inactivation of GSK-3β and thus the accumulation of cytosolic β-catenin. Accumulated β-catenin translocates to the nucleus, where it binds to TCF/LEF transcription factors to activate the transcription of Wnt-responsive genes involved in cell proliferation and differentiation [29]. Various studies have demonstrated an association of TCF7L2 variants with features of metabolic syndrome, including elevated systolic and diastolic blood pressure, increased triglyceride levels, and elevated uric acid [30,31]. Recently, the activation of Wnt/β-catenin signaling in skeletal muscle cells was reported to improve insulin sensitivity by decreasing intramyocellular lipid deposition via downregulation of SREBP-1c [19]. In the present study, we demonstrated that the expression levels of nuclear β-catenin and TCF4, the latter of which is a main transcriptional regulator of Wnt signaling, were decreased in an *in vitro* model of hepatic steatosis. This finding suggests that Wnt signaling could be involved in the regulation of hepatic lipid metabolism.

GLP-1 agonists play important roles in diverse metabolic pathways in hepatocytes, including lipid metabolism, carbohydrate metabolism, and glucose homeostasis [5]. GLP-1 has been reported to regulate Wnt signaling in the pancreas and skeletal muscle [19]. Another study using mouse pancreatic islets found that GLP-1 agonists activated TCF7L2-dependent Wnt signaling by binding to GLP-1 receptor [31]. Moreover, inhibition of Wnt signaling by siRNA-mediated silencing of β-catenin or by the expression of a dominant negative version of TCF7L2 has been shown to decrease both basal and exendin-4-induced β-cell proliferation [31]. We found that the expression levels of β-catenin and TCF4 in exendin-4-treated hepatocytes were increased, and that the effect of exendin-4 on β-catenin expression was impaired upon treatment with the β-catenin inhibitor IWR-1 (S2 Fig). In addition, the blunted effects of exendin-4 in the regulation of lipid accumulation and in the expression of lipogenic genes were observed in hepatocytes treated with IWR-1 (S2 Fig). These data suggest that the protective effects of exendin-4 on hepatic lipid accumulation could be mediated by β-catenin. However, further studies are needed to clarify the precise role of Wnt/β-catenin signaling in hepatic lipogenesis and the mechanism of exendin-4-mediated regulation of β-catenin. Additionally, our study did have a few limitations. HepG2 cell lines are available for mechanism-based testing of drug-induced hepatotoxicity including steatosis, cholestasis, and necrosis. Nevertheless, since our in vitro model was a cancer cell line, additional studies in normal hepatocytes are required.

In conclusion, the present study demonstrated that exendin-4 increases the expression of β-catenin and that inhibition of β-catenin increases the expression of lipogenic transcription factors. Therefore, exendin-4 may inhibit hepatic lipogenesis by activating the Wnt/β-catenin pathway, thus having a potentially beneficial role as a medical treatment for fatty liver disease.

## Supporting Information

S1 Fig Exendin-4 induces active β-catenin signaling in AML12 cells. (EPS)

S2 Fig IWR-1 attenuates the effect of exendin-4 on the expression of β-catenin and lipogenic genes in HepG2 cells. (EPS)
